# Prevalence, Awareness, Treatment and Control of Diabetes Mellitus in a Chinese Population

**DOI:** 10.1371/journal.pone.0153791

**Published:** 2016-04-20

**Authors:** Jiqiang Yue, Xuhua Mao, Kun Xu, Lingshuang Lü, Sijun Liu, Feng Chen, Jianming Wang

**Affiliations:** 1 Department of Social Medicine and Health Education, School of Public Health, Nanjing Medical University, Nanjing, 211166, China; 2 Department of Clinical Laboratory, Yixing People’s Hospital, Wuxi, 214200, China; 3 Department of Epidemiology, School of Public Health, Nanjing Medical University, Nanjing, 211166, China; 4 The Innovation Center for Social Risk Governance in Health, School of Public Health, Nanjing Medical University, Nanjing, 211166, China; Harvard Medical School, UNITED STATES

## Abstract

**Objective:**

The purpose of this study is to evaluate the prevalence, awareness, treatment and glycemic control of diabetes mellitus (DM) in a Chinese population. The findings from this study are expected to offer scientific evidence to better prevent and control the growing number of reported and untreated cases.

**Methods:**

A cross-sectional survey was conducted in Jiangsu, China. We recruited permanent residents over 18 years of age from eight towns in Jintan (JT) and six towns in Yangzhong (YZ) using a three-stage stratified cluster sampling method. The rates of DM prevalence, awareness, treatment and control as well as their related factors were analyzed.

**Results:**

A total number of 15404 people were entered into the analysis. The DM prevalence, awareness, treatment and control rates were 7.31%, 58.35%, 51.87% and 14.12%, respectively. Multivariable logistic regression analysis showed that being female was positively related to prevalence (OR = 1.21, 95% CI: 1.07–1.37), awareness (OR = 1.52, 95% CI: 1.19–1.93), treatment (OR = 1.48, 95% CI: 1.17–1.88) and control (OR = 1.87, 95% CI: 1.30–2.67) of DM. Having a family history of diabetes was significantly correlated with DM risk (OR = 1.86, 95% CI: 1.37–2.54) and increased awareness (OR = 3.12, 95% CI: 2.19–4.47), treatment (OR = 3.47, 95% CI: 2.45–4.90) and control (OR = 1.81, 95% CI: 1.22–2.68) of DM. Former smoking status (OR = 1.82, 95% CI: 1.23–2.71), overweight (OR = 2.11, 95% CI: 1.72–2.60) and obesity (OR = 3.46, 95% CI: 2.67–4.50) were related to the risk of DM. Additionally, we found current drinking status to be positively correlated with DM risk (OR = 1.30, 95% CI: 1.01–1.66) and negatively correlated with DM awareness (OR = 0.41, 95% CI: 0.29–0.59) and treatment (OR = 0.41, 95% CI: 0.29–0.59). Our study highlights the high prevalence and inadequate awareness, treatment and control of DM in the Chinese population.

**Conclusions:**

Management and prevention of DM-related complications should be considered an essential strategy by governments and society. This study assessed the reasons why DM has been increasing and established the first step in determining where to start regarding preventative methods.

## Introduction

The prevalence of diabetes mellitus (DM) has been increasing rapidly worldwide. Although the methodologies used in previous studies have lacked consistency and some studies have had controversial findings [1, 2], China is still considered to have the largest number of DM patients in the world, together with India [3]. Nearly one million new DM cases are reported in China every year [4]. From 1979 through 2012, the prevalence of DM in China has increased significantly; however, there have been no obvious improvements in DM awareness [5]. In 2008, the direct medical cost of diabetes in China reached $9.1 billion [6]. The International Diabetes Federation (IDF) reported that 13% of China’s health expenditures could be attributed to DM management [7]. In light of these numbers, it is clear that there is an urgent need to undertake concerted efforts and implement national programs aimed at the prevention, management and surveillance of DM.

The increasing prevalence of DM has led to tremendous increases in health care costs, in the treatment of the disease and in the management of DM-related complications [8, 9]. Common complications include both microvascular (neuropathy, nephropathy and retinopathy) and macrovascular disorders (cardiovascular disease, stroke and peripheral vascular disease) [5]. Numerous studies have indicated that the burden of DM may be reduced through appropriate interventions [10]. Maintaining blood glucose at a normal level can significantly lower the risk of DM-related complications, causing a delay in disease progression [11–13]. Evaluating the risk factors in a specific population is an essential first step to developing specific intervention strategies [14].

In this study, we used a cross-sectional survey to estimate the burden of DM in the rural communities of Jiangsu province, China. This survey will provide scientific evidence regarding the integrated prevention and control strategies for chronic diseases in the communities and will provide comprehensive and advanced suggestions to improve the level of health and quality of life of DM patients.

## Materials and Methods

### Study sites and study population

Jiangsu is one of the most developed provinces in China, with a population of 79 million and an area of 102600 square kilometers. We selected Jintan (JT) county and Yangzhong (YZ) county from Jiangsu province as the study sites. Together, the counties host populations of 550000 and 340000, respectively. The authors carried out a community-based cross-sectional survey of residents aged 18 years and over. Residents were required to have lived in the research sites for more than 6 of the previous 12 months in the year 2013.

A three-stage stratified cluster sampling method was applied in this survey. In the first stage, we selected JT and YZ as the study settings. Then, of the eight towns in JT and the six towns in YZ, one village was randomly selected from each town for a total of 14 villages as research sites. The local residents in these villages were recruited as the study subjects. To estimate the sample size, the authors initially began with an estimated prevalence of DM (JT: p = 6%, YZ: p = 8%); next, the tolerance level was set at d = 0.1×p, and the significance level at = 0.05. Referring to the formula n = 400*q/p, the sample sizes in JT and YZ were expected to be 6267 and 4600, respectively. Taking the sampling error into consideration, the authors determined the design efficiency (*deff*) to be 1.5 and then estimated the non-response rate to be 20%. Using this method, the calculated expected sample size was 10341 in JT and 7590 in YZ. A final total of 17949 permanent residents aged over 18 years in sampled villages were recruited as the study population, 15566 of whom answered the questionnaire. After removing the subjects who did not provide the necessary information, a final population of 15404 people was analyzed, with an overall response rate of 85.8% (15404/17949). This project was approved by the Institutional Review Board of Nanjing Medical University. Written informed consent was obtained from all participants.

### Data collection

Trained town hospital staff interviewers administered a questionnaire to all participants. Information on demographics (e.g., age, gender, residential area), socioeconomic status (e.g., education level, marital status), behaviors (e.g., cigarette smoking, alcohol use, physical activity) and history of common chronic diseases (e.g., hypertension, DM, heart disease, stroke, cancer) was collected. Blood pressure was measured by using a mercury sphygmomanometer. Respondents were measured three times in a relaxed state. Vacuum blood collection tubes with volumes of 3–5 ml were used to collect fasting blood. Blood samples were centrifuged and separated in time and were then stored according to standards. We measured blood glucose, lipids, alanine aminotransferase (ALT) and creatinine (renal function) concentrations.

### Definitions

In this study, subjects whose casual plasma glucose value was greater than 7.0 mmol/L without a history of DM were defined as possible new DM cases. Those who had been previously diagnosed with DM were defined as prevalent cases. The possible new cases were referred to town-level or above hospitals to ensure a formal diagnosis. “Awareness of DM” was defined in this paper as participants with DM who self-reported a previous diagnosis of DM by a physician. “Treatment of DM” was determined by whether participants fulfilled at least one of the following options: (a) taking oral hypoglycemic medications, (b) injecting insulin, or (c) using other non-pharmacological treatments such as diet control and exercise to manage high glucose levels. “Control of DM” was defined as a fasting plasma glucose lower than 7.0 mmol/L [15]. Body Mass Index (BMI) was calculated as weight (Kg) divided by height (m^2^) and was classified as under/normal weight if BMI < 24 kg/m^2^, overweight if BMI was between 24 and 28 kg/m^2^ and obese if BMI ≥ 28 kg/m^2^ [16]. “Low education level” was defined as participants who were illiterate. “Medium education level” was defined as those who had achieved less than a high school education. “High education level” was defined by those who had completed high school education and/or higher.

### Statistical analysis

The Data Input Working Group adopted a unified database Epidata 3.1 (Denmark) for data entry. All statistical analyses were performed using Stata 12.0 (College Station, TX, USA). The prevalence, awareness, treatment and control of DM were described as proportions. Student’s t-test (for continuous variables) and the χ^2^ test (for categorical variables) were used to analyze the differences in demographic variables and potential risk factors between groups. A multivariate logistic regression model was used to assess the risk factors by adjusting for potential confounders. Two-tailed *P*-values less than 0.05 were considered statistically significant.

## Results

### General characteristics

A total number of 15404 people were entered into the analysis. There were 7293 (47.34%) men and 8111 (52.66%) women participating in the study. The average age was 54.44 (±15.76) years. As shown in Table 1, 16.39% of the responding population was illiterate, whereas the well-educated people who had a high school education or above composed 16.43%. Additionally, 74.61% of the respondents had never smoked, and 3.89% of the respondents were former smokers. Regarding alcohol use, 80.00% of the respondents never drank alcohol, 1.17% of the respondents were former drinkers, and 18.83% of the population currently drank alcohol. There were 8971 study subjects in JT and 6433 subjects in YZ.

**Table 1:** Basic characteristics of the study subjects.

Variables	Total (n = 15404), N(%)
**Age (years)**	
**18-**	**2732(17.74)**
**40-**	**6427(41.72)**
**60-**	**5487(35.62)**
**80-**	**758(4.92)**
**Gender**	
**Male**	**7293(47.34)**
**female**	**8111(52.66)**
**Education**	
**Low**	**2524(16.39)**
**Middle**	**10349(67.18)**
**High**	**2531(16.43)**
**Smoking**	
**Never**	**11479(74.61)**
**Former**	**598(3.89)**
**Current**	**3309(21.50)**
**Drinking**	
**Never**	**12316(80.00)**
**Former**	**180(1.17)**
**Current**	**2899(18.83)**
**City**	
**JT**	**8971(58.24)**
**YZ**	**6433(41.76)**

Abbreviations: JT, Jintan; YZ, Yangzhong

### Prevalence of DM

The overall DM prevalence was 7.31% (1126/15404, 95% CI: 6.90%-7.73%) for both genders, 6.71% (489/7293, 95% CI: 6.14%-7.30%) for males and 7.85% (637/8111, 95% CI: 7.28%-8.46%) for females. The overall glycemic level of the study population was 5.66±1.86 mmol/L. The glycemic levels of men and women were 5.64±1.87 mmol/L and 5.67±1.85 mmol/L, respectively. In the age group of 60- to 80-year-olds, the DM prevalence in women (12.33%, 95% CI:11.13%-13.61%) was significantly higher than that of men (9.29%, 95% CI: 8.22%-10.44%). The factors associated with DM prevalence included gender, age, education, BMI, family history, smoking, intensity of physical labor and exercise (Table 2).

**Table 2:** Factors associated with DM prevalence.

Variables	Non-Diabetic, N(%)	Diabetic, N(%)	aOR(95% CI)^#^	aP^#^
**Gender**				
**Male**	**6804(93.29)**	**489(6.71)**	**1**	
**Female**	**7474(92.15)**	**637(7.85)**	**1.21(1.07–1.37)**	**0.003**
**Age (years)**				
**18-**	**2688(98.39)**	**44(1.61)**	**1**	
**40-**	**6004(93.42)**	**423(6.58)**	**4.29(3.13–5.87)**	**<0.001**
**60-**	**4893(89.17)**	**594(10.83)**	**7.44(5.46–10.15)**	**<0.001**
**80-**	**693(91.42)**	**65(8.58)**	**5.72(3.86–8.46)**	**<0.001**
**Education**				
**Low**	**2241(88.79)**	**283(11.21)**	**1**	
**Middle**	**9613(92.89)**	**736(7.11)**	**0.80(0.68–0.94)**	**0.007**
**High**	**2424(95.77)**	**107(4.23)**	**0.88(0.67–1.15)**	**0.339**
**BMI**				
**<24**	**9366(94.76)**	**518(5.24)**	**1**	
**24-**	**3787(89.95)**	**423(10.05)**	**1.89(1.65–2.16)**	**<0.001**
**28-**	**1125(85.88)**	**185(14.12)**	**2.79(2.33–3.34)**	**<0.001**
**Family history of DM**				
**No**	**13436(93.61)**	**917(6.39)**	**1**	
**Yes**	**842(80.11)**	**209(19.89)**	**3.87(3.27–4.59)**	**<0.001**
**Drinking**				
**Never**	**11401(92.57)**	**915(7.43)**	**1**	
**Former**	**162(90.00)**	**18(10.00)**	**1.16(0.70–1.91)**	**0.570**
**Current**	**2706((93.34)**	**193(6.66)**	**0.83(0.69–0.99)**	**0.047**
**Smoking**				
**Never**	**10629(92.60)**	**850(7.40)**	**1**	
**Former**	**533(89.13)**	**65(10.87)**	**1.45(1.08–1.93)**	**0.012**
**Current**	**3100(93.68)**	**209(6.32)**	**0.87(0.72–1.06)**	**0.166**
**Physical labor intensity**				
**Low**	**7029(91.35)**	**666(8.65)**	**1**	
**Medium**	**5879(94.08)**	**370(5.92)**	**0.73(0.63–0.83)**	**<0.001**
**High**	**1337(93.82)**	**88(6.18)**	**0.74(0.58–0.94)**	**0.014**
**Exercise**				
**No**	**13184(93.04)**	**987(6.96)**	**1**	
**Yes**	**1034(88.60)**	**133(11.40)**	**1.71(1.41–2.08)**	**<0.001**
**Average daily sleep (hours)**				
**<6**	**8276(92.27)**	**693(7.73)**	**1**	
**6–8**	**1920(93.16)**	**141(6.84)**	**0.91(0.75–1.10)**	**0.325**
**8-**	**4082(93.32)**	**292(6.68)**	**1.02(0.88–1.17)**	**0.835**
**Residential area**				
**JT**	**8306(92.59)**	**665(7.41)**	**1**	
**YZ**	**5972(92.83)**	**461(7.17)**	**1.08(0.95–1.22)**	**0.228**

^#^Adjusted for age and sex;

Abbreviations: OR, odds ratio; CI, confidence interval; JT, Jintan; YZ, Yangzhong

### Risk of DM

To avoid the prevalence-incidence bias (Neyman bias), we further performed a risk analysis by using only the newly detected DM patients as the cases. As shown in Table 3, older age, overweight or obesity, a family history of diabetes, a history of former cigarette smoking, current drinking status and residence in JT county turned out to be significant risk factors (OR>1) of DM. Medium/high educational levels and more than eight hours of daily sleep were significant protective factors (OR<1).

**Table 3:** Factors associated with the possible risk of DM.

Variables	Non-Diabetic, N(%)	Diabetic, N(%)*	aOR(95% CI)^#^	aP^#^
**Gender**				
**Male**	**6804(96.70)**	**232(3.30)**	**1**	
**Female**	**7474(96.93)**	**237(3.07)**	**0.94(0.78–1.14)**	**0.545**
**Age (years)**				
**18-**	**2688(99.04)**	**26(0.96)**	**1**	
**40-**	**6004(96.92)**	**191(3.08)_**	**3.29(2.18–4.97)**	**<0.001**
**60-**	**4893(95.55)**	**228(4.45)**	**4.81(3.20–7.24)**	**<0.001**
**80-**	**693(96.65)**	**24(3.35)**	**3.58(2.04–6.28)**	**<0.001**
**Education**				
**Low**	**2241(95.28)**	**111(4.72)**	**1**	
**Middle**	**9613(96.83)**	**315(3.17)**	**0.73(0.57–0.94)**	**0.014**
**High**	**2424(98.26)**	**43(1.74)**	**0.63(0.42–0.96)**	**0.030**
**BMI**				
**<24**	**9366(97.91)**	**200(2.09)**	**1**	
**24-**	**3787(95.41)**	**182(4.59)**	**2.11(1.72–2.60)**	**<0.001**
**28-**	**1125(92.82)**	**87(7.18)**	**3.46(2.67–4.50)**	**<0.001**
**Family history of DM**				
**No**	**13436(96.95)**	**422(3.05)**	**1**	
**Yes**	**842(94.71)**	**47(5.29)**	**1.86(1.37–2.54)**	**<0.001**
**Drinking**				
**Never**	**11401(97.06)**	**345(2.94)**	**1**	
**Former**	**162(95.86)**	**7(4.14)**	**1.20(0.55–2.60)**	**0.652**
**Current**	**2706(95.86)**	**117(4.14)**	**1.30(1.01–1.66)**	**0.039**
**Smoking**				
**Never**	**10629(97.01)**	**328(2.99)**	**1**	
**Former**	**533(94.00)**	**34(6.00)**	**1.82(1.23–2.71)**	**0.003**
**Current**	**3100(96.69)**	**106(3.31)**	**1.05(0.80–1.37)**	**0.743**
**Physical labor intensity**				
**Low**	**7029(96.66)**	**243(3.34)**	**1**	
**Medium**	**5879(97.08)**	**177(2.92)**	**0.91(0.74–1.12)**	**0.380**
**High**	**1337(96.46)**	**49(3.54)**	**1.04(0.75–1.44)**	**0.801**
**Exercise**				
**No**	**13184(96.90)**	**422(3.10)**	**1**	
**Yes**	**1034(95.74)**	**46(4.26)**	**1.40(1.02–1.91)**	**0.035**
**Average daily sleep (hours)**				
**<6**	**8276(96.32)**	**316(3.68)**	**1**	
**6–8**	**1920(97.26)**	**54(2.74)**	**0.76(0.56–1.01)**	**0.062**
**8-**	**4082(97.63)**	**99(2.37)**	**0.73(0.58–0.92)**	**0.007**
**Residential area**				
**JT**	**8306(96.41)**	**309(3.59)**	**1**	
**YZ**	**5972(97.39)**	**160(2.61)**	**0.79(0.65–0.96)**	**0.017**

*Based on newly detected DM cases;

^#^Adjusted for age and sex;

Abbreviations: OR, odds ratio; CI, confidence interval; JT, Jintan; YZ, Yangzhong

### Awareness of DM

Of all subjects diagnosed with DM, 58.35% (657/1126, 95% CI: 55.40%-61.25%) already knew about their diabetic status. The awareness rate was 52.56% (257/489, 95% CI: 48.02%-57.06%) for men and 62.79% (400/637, 95% CI: 58.91%-66.56%) for women. Participants who were female, were older, had a high level of education, had a diabetic family history, slept more than eight hours daily and lived in YZ were more likely to be well informed of their diabetic status (OR>1). However, current alcohol consumption and medium/high-intensity physical labor increased the risk of low awareness of diabetic status (OR<1) (Table 4).

**Table 4:** Factors associated with diabetes mellitus awareness.

Variables	Unaware, N(%)	Aware, N(%)	aOR(95% CI)^#^	aP^#^
**Gender**				
**Male**	**232(47.44)**	**257(52.56)**	**1**	
**Female**	**237(37.21)**	**400(62.79)**	**1.52 (1.19–1.93)**	**0.001**
**Age (years)**				
**18-**	**26(59.09)**	**18(40.91)**	**1**	
**40-**	**191(45.15)**	**232(54.85)**	**1.74(0.92–3.28)**	**0.087**
**60-**	**228(38.38)**	**366(61.62)**	**2.29(1.22–4.28)**	**0.010**
**80-**	**24(36.92)**	**41(63.08)**	**2.46(1.11–5.41)**	**0.025**
**Education**				
**Low**	**111(39.22)**	**172(60.78)**	**1**	
**Middle**	**315(42.80)**	**421(57.20)**	**1.17(0.86–1.60)**	**0.318**
**High**	**43(40.19)**	**64(59.81)**	**1.70(1.01–2.84)**	**0.044**
**BMI**				
**<24**	**200(38.61)**	**318(61.39)**	**1**	
**24-**	**182(43.03)**	**241(56.97)**	**0.87(0.67–1.14)**	**0.313**
**28-**	**87(47.03)**	**98(52.97)**	**0.73(0.52–1.03)**	**0.078**
**Family history of DM**				
**No**	**422(46.02)**	**495(53.98)**	**1**	
**Yes**	**47(22.49)**	**162(77.51)**	**3.12(2.19–4.47)**	**<0.001**
**Drinking**				
**Never**	**345(37.70)**	**570(62.30)**	**1**	
**Former**	**7(38.89)**	**11(61.11)**	**0.94(0.36–2.50)**	**0.904**
**Current**	**117(60.62)**	**76(39.38)**	**0.41(0.29–0.59)**	**<0.001**
**Smoking**				
**Never**	**328(38.59)**	**522(61.41)**	**1**	
**Former**	**34(52.31)**	**31(47.69)**	**0.66(0.38–1.15)**	**0.143**
**Current**	**106(50.72)**	**103(49.28)**	**0.74(0.51–1.07)**	**0.108**
**Physical labor intensity**				
**Low**	**243(36.49)**	**423(63.51)**	**1**	
**Medium**	**177(47.84)**	**193(52.16)**	**0.68(0.52–0.89)**	**0.006**
**High**	**49(55.68)**	**39(44.32)**	**0.51(0.32–0.81)**	**0.004**
**Exercise**				
**No**	**422(42.76)**	**565(57.24)**	**1**	
**Yes**	**46(34.59)**	**87(65.41)**	**1.42(0.97–2.09)**	**0.072**
**Average daily sleep (hours)**				
**<6**	**316(45.60)**	**377(54.40)**	**1**	
**6–8**	**54(38.30)**	**87(61.70)**	**1.44(0.98–2.10)**	**0.058**
**8-**	**99(33.90)**	**193(66.10)**	**1.77(1.32–2.37)**	**<0.001**
**Residential area**				
**JT**	**309(46.47)**	**356(53.53)**	**1**	
**YZ**	**160(34.71)**	**301(65.29)**	**1.72(1.34–2.21)**	**<0.001**

^#^Adjusted for age and sex;

Abbreviations: OR, odds ratio; CI, confidence interval; JT, Jintan; YZ, Yangzhong

### Treatment of DM

The DM treatment rate was 51.87% (584/1126, 95% CI: 48.90%-54.82%) in individuals with DM and 88.89% (584/657, 95% CI: 86.23%-91.19%) in individuals who already knew their diabetic status. The treatment rates were 46.22% (226/489, 95% CI: 41.73%-50.75%) for males and 56.20% (358/637, 95% CI: 52.25%-60.10%) for females. People who were female, elderly, and residents with a diabetic family history were more inclined to be treated; however, people who currently consumed alcohol and those who had medium/high-intensity physical labor were less inclined to be treated (Table 5).

**Table 5:** Factors associated with diabetes mellitus treatment.

Variables	Untreated, N(%)	Treated, N(%)	aOR(95% CI)^#^	aP^#^
**Gender**				
**Male**	**263(53.78)**	**226(46.22)**	**1**	
**Female**	**279(43.80)**	**358(56.20)**	**1.48(1.17–1.88)**	**0.001**
**Age (years)**				
**18-**	**33(75.00)**	**11(25.00)**	**1**	
**40-**	**217(51.30)**	**206(48.70)**	**2.83(1.39–5.77)**	**0.004**
**60-**	**259(42.60)**	**335(56.40)**	**3.84(1.90–7.78)**	**<0.001**
**80-**	**33(50.77)**	**32(49.23)**	**2.90(1.25–6.73)**	**0.013**
**Education**				
**Low**	**130(45.94)**	**153(54.06)**	**1**	
**Middle**	**359(48.78)**	**377(51.22)**	**1.17(0.86–1.60)**	**0.307**
**High**	**53(49.53)**	**54(50.47)**	**1.53(0.92–2.54)**	**0.100**
**BMI**				
**<24**	**236(45.56)**	**282(54.44)**	**1**	
**24-**	**209(49.41)**	**214(50.59)**	**0.91(0.70–1.18)**	**0.471**
**28-**	**97(52.43)**	**88(47.57)**	**0.80(0.56–1.12)**	**0.192**
**Family history of DM**				
**No**	**487(53.11)**	**430(46.89)**	**1**	
**Yes**	**55(26.32)**	**154(73.68)**	**3.47(2.45–4.90)**	**<0.001**
**Drinking**				
**Never**	**406(44.37)**	**509(55.63)**	**1**	
**Former**	**8(44.44)**	**10(55.56)**	**0.95(0.37–2.49)**	**0.923**
**Current**	**128(66.32)**	**65(33.68)**	**0.41(0.29–0.59)**	**<0.001**
**Smoking**				
**Never**	**386(45.41)**	**464(54.59)**	**1**	
**Former**	**37(56.92)**	**28(43.08)**	**0.71(0.41–1.24)**	**0.227**
**Current**	**117(55.98)**	**92(44.02)**	**0.78(0.54–1.13)**	**0.188**
**Physical labor intensity**				
**Low**	**287(43.09)**	**379(56.91)**	**1**	
**Medium**	**202(54.59)**	**168(45.41)**	**0.68(0.52–0.89)**	**0.005**
**High**	**52(59.09)**	**36(40.91)**	**0.56(0.35–0.89)**	**0.015**
**Exercise**				
**No**	**481(48.73)**	**506(51.27)**	**1**	
**Yes**	**56(42.11)**	**77(57.89)**	**1.28(0.89–1.86)**	**0.187**
**Average daily sleep (hours)**				
**<6**	**345(49.78)**	**348(50.22)**	**1**	
**6–8**	**64(45.39)**	**77(54.61)**	**1.27 (0.88–1.84)**	**0.203**
**8-**	**133(45.55)**	**159(54.45)**	**1.29 (0.97–1.70)**	**0.078**
**Residential area**				
**JT**	**336(50.53)**	**329(49.47)**	**1**	
**YZ**	**206(44.69)**	**255(55.31)**	**1.33 (1.04–1.70)**	**0.021**

^#^Adjusted for age and sex;

Abbreviations: OR, odds ratio; CI, confidence interval; JT, Jintan; YZ, Yangzhong

### Control of DM

The DM control rate was 14.12% (159/1126, 95% CI: 12.14%-16.29%) among all patients with DM and 27.23% (159/584, 95% CI: 23.65%-31.03%) among the treated patients. The control rates were 10.02% (49/489, 95% CI: 7.50%-13.03%) for men and 17.27% (110/637, 95% CI: 14.41%-20.43%) for women. Female gender and a family history of diabetes were associated with a higher likelihood of having a controlled glycemic level, whereas people involved in high-intensity physical labor were less likely to have their blood glucose controlled (Table 6).

**Table 6:** Factors associated with diabetes mellitus control.

Variables	Uncontrolled, N(%)	Controlled, N(%)	aOR(95% CI)^#^	aP^#^
**Gender**				
**Male**	**440(89.98)**	**49(10.02)**	**1**	
**Female**	**527(82.73)**	**110(17.27)**	**1.87 (1.30–2.67)**	**0.001**
**Age (years)**				
**18-**	**39(88.64)**	**5(11.36)**	**1**	
**40-**	**376(88.89)**	**47(11.11)**	**0.95 (0.36–2.55)**	**0.926**
**60-**	**497(83.67)**	**97(16.23)**	**1.48 (0.57–3.87)**	**0.423**
**80-**	**55(84.62)**	**10(15.38)**	**1.40 (1.30–2.67)**	**0.571**
**Education**				
**Low**	**237(83.75)**	**46(16.25)**	**1**	
**Middle**	**635(86.28)**	**101(13.72)**	**1.15 (0.76–1.74)**	**0.516**
**High**	**95(88.79)**	**12(11.21)**	**1.21 (0.57–2.58)**	**0.615**
**BMI**				
**<24**	**454(87.64)**	**64(12.36)**	**1**	
**24-**	**357(84.40)**	**66(15.60)**	**1.38 (0.94–2.00)**	**0.097**
**28-**	**156(84.32)**	**29(15.68)**	**1.35 (0.83–2.18)**	**0.227**
**Family history of DM**				
**No**	**801(87.35)**	**116(12.65)**	**1**	
**Yes**	**166(79.43)**	**43(50.57)**	**1.81 (1.22–2.68)**	**0.003**
**Drinking**				
**Never**	**776(84.81)**	**139(15.19)**	**1**	
**Former**	**16(88.89)**	**2(11.11)**	**0.92 (0.20–4.19)**	**0.919**
**Current**	**175(90.67)**	**18(9.33)**	**0.82 (0.46–1.46)**	**0.496**
**Smoking**				
**Never**	**718(84.47)**	**132(15.53)**	**1**	
**Former**	**60(92.31)**	**5(7.69)**	**0.67 (0.25–1.81)**	**0.425**
**Current**	**187(89.47)**	**22(10.53)**	**1.00 (0.56–1.81)**	**0.992**
**Physical labor intensity**				
**Low**	**562(84.38)**	**104(15.62)**	**1**	
**Medium**	**319(86.22)**	**51(13.78)**	**1.00 (0.68–1.46)**	**0.992**
**High**	**84(95.45)**	**4(4.55)**	**0.32 (0.11–0.91)**	**0.033**
**Exercise**				
**No**	**844(85.51)**	**143(14.49)**	**1**	
**Yes**	**117(87.97)**	**16(12.03)**	**0.83 (0.47–1.44)**	**0.506**
**Average daily sleep (hours)**				
**<6**	**585(84.42)**	**108(15.58)**	**1**	
**6–8**	**124(87.94)**	**17(12.06)**	**0.80 (0.46–1.40)**	**0.435**
**8-**	**258(88.36)**	**34(11.64)**	**0.75 (0.49–1.13)**	**0.171**
**Residential area**				
**JT**	**562(84.51)**	**103(15.49)**	**1**	
**YZ**	**405(87.85)**	**56(12.15)**	**0.78 (0.55–1.11)**	**0.172**

^#^Adjusted for age and sex;

Abbreviations: OR, odds ratio; CI, confidence interval; JT, Jintan; YZ, Yangzhong

## Discussion

Jiangsu’s prospering economy and strong academic environment is well known throughout China. Despite this wealth, issues concerning the prevalence, awareness, treatment, control and related risk factors of DM have not received sufficient attention. The overall prevalence of DM in this study population was higher than those found in previous studies conducted in other areas of China (5.49% from Hu et al. and 6.41% from Li et al.)[5, 17] and lower than the figures reported in other countries such as in America [18], Thailand [19], Malaysia [20] and India [21, 22] (Table 7).

**Table 7:** Comparison of four DM-correlated rates in different areas.

Author	Age	N	Area	Year	Prevalence, %	Awareness, %	Treatment, %	Control, %
**Current study**	**>18**	**15404**	**China**	**2013**	**7.31**	**58.35**	**51.87**^b^^2^**; 88.89**^a^^2^	**14.12**^d^**; 27.23**^c^
**Faith et al. [**23**]**	**25–64**	**1255**	**Africa**	**2007**	**11.50**	**54**	**NA**	**<25**^c^
**Hu et al. [**17**]**	**35–74**	**15236**	**China**	**2008**	**5.49**	**23.66**	**85.22**^a^^2^	**35.00**^e^
**Porapakkham et al. [**19**]**	**>60**	**19374**	**Thailand**	**2008**	**14.00**	**58.80**	**NA**	**26.40**
**Rampal et al. [**20**]**	**≥30**	**7683**	**Malaysia**	**2010**	**15.20**	**45.00**	**42.70**^b^	**25.10**^c^
**Sims et al. [**18**]**		**4303**	**America**	**2011**	**Female: 19.6**	**90.00**	**86.80**	**39.20**
					**Male: 15.9**	**88.20**	**84.40**	**35.90**
**Singh et al. [**22**]**	**≥60**	**474**	**India**	**2012**	**18.80**	**1/3**	**2/3**^a^	**3/4**^c^
**Yang et al. [**24**]**	**≥35**	**14122**	**China**	**2012**	**Han: 9.26**	**53.00**	**26.70**^b^^1^	**10.40**^d^
					**Uygurs: 6.23**	**35.80**	**7.30**^b^^1^	**3.13**^d^
					**Kazak: 3.65**	**23.80**	**6.30**^b^^1^	**1.40**^d^
**Li et al. [**5**]**	**NA**	**NA**	**China**	**1979–2012**	**6.41**	**45.81**	**42.54**	**20.87**
**Gupta et al. [**21**]**	**≥20**	**6198**	**India**	**2014**	**15.70**	**72.40**	**54.10**	**39.60**
**Wang et al. [**15**]**	**18–79**	**1854**	**China**	**2014**	**NA**	**64.10**	**52.90**	**44.20**
**Rahman et al. [**25**]**	**≥35**	**7786**	**Bangladesh**	**2015**	**9.20**	**41.20**	**36.90**^b^	**14.20**^d^

^a.^ The proportion of treated DM patients of the DM patients who were aware of their diabetic condition.

^b.^ The proportion of treated DM patients of all DM patients.

^c.^ The proportion of DM patients whose glycemic level was controlled among all treated DM patients.

^d.^ The proportion of DM patients whose glycemic level was controlled among all DM patients.

^e.^ The proportion of DM patients whose glycemic level was controlled among all DM patients who were aware of their diabetic status.

^1.^ Patients only took drug treatment

^2.^ Patients took drug and non-drug treatment.

The DM awareness and treatment rates of the current study were higher but the control rates lower than those found in other studies conducted in China, as reported by Hu et al. [17], Li et al. [24] and Wang et al. [15]. After age-sex adjustment, our study found that a high level of education was not only a protective factor for developing DM but also a factor that increased DM awareness. Similar reports from China [17] have also found that people with an education level higher than high school (5.28%) were less likely to be diabetic than were those with lower educational levels (5.55%).

Obesity is a major contributor to chronic disease [26]. We observed that overweight and obesity were strongly associated with the risk of DM. Similar results regarding excess weight have been found in other areas, such as the Seychelles [23], Jordan [27] and Saudi Arabia [28]. Previous intervention studies have shown that diet and exercise therapy have good effectiveness and low costs, can be easily accepted and have low rates of negative consequences [29, 30].

A significant difference in DM awareness was mainly observed between different genders. Women tended to be more aware of their diabetic status, which was consistent with previous reports [15, 18, 24, 31]. Our study also found that women were more likely to take DM treatment and to have controlled blood glucose levels. This could have been due to the nature of women, as women are more emotional and more sensitive to changes in their own physical condition, whereas Chinese men, in most cases, must work very hard to provide financial support and rarely have the time or inclination to take care of their own health.

Family history was found to be a risk factor for DM as well as a protective factor for DM awareness, treatment and control. There could be several reasons that explain this phenomena. The high prevalence of DM within the same family could have originated from the fact that family members share the same hereditary material, or it could be explained by the fact that younger generations may have inherited the same unhealthy and diabetes-causing dietary systems of their elders. At the same time, the family members of the diabetic patients tended to be more conscious and more experienced with controlling DM.

Sleep deprivation (defined here as less than 8 hours of sleep a night) has previously been proven to be correlated to impaired fasting glucose [32], central fat distribution, and increased insulin insensitivity [33]. Our study also found that respondents who reported an average of more than eight hours of sleep every night were less likely to be DM patients. Furthermore, our study found that having over eight hours of sleep a night had a positive correlation with increased DM awareness.

### Strengths of this study

The pre-diagnosed DM patients interviewed in this study may already have possessed a certain degree of understanding of DM. These patients may have previously applied some intervention measures for DM prior to our research. To avoid a prevalence-incidence bias in this study, we performed a risk analysis by only using newly detected DM patients as the cases to reflect a more comprehensive result.

In this study, we systematically analyzed a series of factors related to the prevalence, risk, awareness, treatment and control of DM. As shown in Table 8, some of the factors had similar effects on these DM-related rates, but some had different effects. Individualized intervention strategies are recommended to achieve a more effective prevention and control of DM.

**Table 8:** Factors related to the risk, awareness, treatment and control of diabetes mellitus.

Factors	Risk	Awareness	Treatment	Control
**Women (vs. men)**	**↓NS**	↑**	↑**	↑**
**Aged 40~60 years (vs. <40)**	↑**	**↑NS**	↑**	**↓NS**
**Aged 60~80 years (vs. <40)**	↑**	↑**	↑**	**↑NS**
**Aged over 80 years (vs. <40)**	↑**	↑*	↑*	**↑NS**
**Middle level of education (vs. low level)**	↓*	**↑NS**	**↑NS**	**↑NS**
**High level of education (vs. low level)**	↓*	↑*	**↑NS**	**↑NS**
**Overweight (vs. normal)**	↑**	**↓NS**	**↓NS**	**↑NS**
**Obesity (vs. normal)**	↑**	**↓NS**	**↓NS**	**↑NS**
**Family history**	↑**	↑**	↑**	↑*
**Former drinking (vs. never drinking)**	**↑NS**	**↓NS**	**↓NS**	**↓NS**
**Current drinking (vs. never drinking)**	↑*	↓**	↓**	**↓NS**
**Former smoking (vs. never smoking)**	↑**	**↓NS**	**↓NS**	**↓NS**
**Current smoking (vs. never smoking)**	**↑NS**	**↓NS**	**↓NS**	**↓NS**
**Middle level of physical labor intensity (vs. low level)**	**↓NS**	↓**	↓**	**↑NS**
**High level of physical labor intensity (vs. low level)**	**↑NS**	↓**	↓*	↓*
**Over 8 hours’ sleeping (vs. <6)**	↓**	↑**	**↑NS**	**↓NS**

NS: non-significant;

*: P<0.05;

**:P<0.01

### Limitations of this study

The findings in this study were subject to several limitations. First, the study was not able to avoid heterogeneity in comparing DM correlated rates with different studies. The treatment rate of DM in our study could have been due to the favorable economic environment and high education level, but it could also have been affected by the different definitions of treatment accepted by different researchers. Our research included drug and non-drug treatments, whereas the others may have only accepted medications as treatment. Second, the study was limited to the villages from JT and YZ counties, which could limit the study’s ability to generalize the findings across the entire population of Jiangsu province. Third, due to the nature of cross-sectional surveys, the paper cannot speculate regarding the causality of DM.

### Implications for policy and clinical practice

The current health care system has been largely built to treat patients with severe and acute conditions [34]. Inadequate attention has been paid to chronic diseases such as DM. Preventing and managing DM should be considered an essential strategy by governments and society, and as such, management measures should be integrated closely into current policies. Intervention programs should certainly be implemented and should focus on intensifying health care particularly for the elderly, strengthening the education of less-educated people, and adopting weight control programs for overweight individuals as well as managing patients who have a family history of DM, smoking habits, alcohol consumption tendencies and physical labor intensities above medium.

## Conclusions

Our study highlighted the high prevalence and inadequate awareness, treatment and control of DM in the Chinese population. Intervention programs to reduce risk behaviors and screening programs for early detection, treatment and control of DM need to be improved. Management and prevention of DM-related complications should be considered an essential strategy by the government and society. This study gained an understanding of why DM has been increasing and made the first step in determining where to begin with preventative methods.
